# The Accuracy of Digital Impressions versus Conventional Impressions in Neonates with Cleft Lip and/or Palate: A Laboratory-Based Study

**DOI:** 10.3390/children11070827

**Published:** 2024-07-06

**Authors:** Jyotsna Unnikrishnan, Mahmoud Bakr, Robert Love, Ghassan Idris

**Affiliations:** 1 School of Medicine and Dentistry, Griffith University, Gold Coast, QLD 4222, Australia; jyotsna.unnikrishnan@griffithuni.edu.au (J.U.); m.bakr@griffith.edu.au (M.B.); r.love@griffith.edu.au (R.L.); 2 Oral Health Service, Metro North Hospital and Health Service, Queensland Children’s Hospital, South Brisbane, QLD 4101, Australia

**Keywords:** cleft lip and palate, digital impressions, neonates, accuracy

## Abstract

Cleft lip and palate (CL/P) are a common congenital craniofacial anomaly requiring precise impressions for effective treatment. Conventional impressions (CIs) pose challenges in neonates with CL/P due to their delicate oral anatomy. While digital impressions (DIs) are increasingly recognised for their potential benefits over conventional methods in dentistry, their accuracy and application in neonates with cleft lip and palate (CL/P) remain underexplored. This study aimed to assess the accuracy of DIs compared to CIs in neonates with CL/P, using alginate and putty materials as references. A laboratory-based case–control study was conducted, utilising soft acrylic models resembling neonatal mouths with CL/P. Alginate and putty impressions were obtained conventionally, while digital impressions were captured using an intraoral scanner (IOS). A total of 42 models were analysed, divided evenly into three groups, with each group comprising 14 models. Superimposition and surface discrepancy analyses were performed to evaluate impression accuracy. The results revealed no statistically significant differences between the digital and conventional impressions in their intra-arch measurements and surface discrepancies. The mean measurement values did not significantly differ among groups, with *p* values indicating no significant variations (*p* > 0.05), confirmed by an analysis of variance. High intra-examiner reliability with Intra Class Coefficient (ICC) values close to 1 indicated consistent measurements over time. The current study demonstrates that DIs are equally accurate as conventional alginate and putty impressions in neonates with cleft lip and palate, offering a viable and less invasive alternative for clinical practise. This advancement holds promise for improving the treatment planning process and enhancing patient comfort, particularly in vulnerable neonatal populations. Further research is warranted to explore the clinical implications and factors affecting DI accuracy in this population.

## 1. Introduction

Cleft lip and/or palate (CL/P) are a highly prevalent congenital anomaly affecting neonates and necessitate specialised, multidisciplinary, and meticulous care from an early age [1,2]. The orthodontic management of neonates with CL/P begins soon after birth with Naso Alveolar Moulding (NAM), with the accuracy of oral impressions serving as a fundamental aspect in treatment planning and intervention [3]. The World Health Organisation recommends study models to be recorded at various stages; soon after birth, before the lip’s surgical repair, and at 5, 10, 18, and 20 years of age [4]. Hence, in neonates, an oral impression is one of the first procedures undertaken as a diagnostic aid for constructing oral appliances, mainly for pre-operative orthopaedic preparation.

Conventional impression (CI) techniques using alginate or rubber-based materials have proven challenging in neonates due to the anatomical complexities and potential risks associated with airway obstruction during impression procedures [5]. Due to the constricted airway of infants, CI procedures may result in a reduction in oxygen saturation levels, and it is recommended that a reduction of more than 5 percent should receive an additional oxygen supply [6]. Consequently, the impression procedure for neonates with CL/P is mostly carried out within a hospital environment due to the elevated risk of respiratory complications, as the procedure can also potentially obstruct the airway or elicit a gag reflex, necessitating immediate medical intervention [3]. Moreover, the plaster models from CI have the added disadvantage of being brittle, cumbersome, and heavy, which makes their storage, transfer, and retrieval difficult. Additionally, plaster models are susceptible to dimensional changes [7].

Recently, the production of a digital impression (DI), facilitated by an intraoral scanner (IOS), has been introduced to be used at various stages of cleft care [8,9,10,11,12,13,14,15,16,17,18,19,20]. DI is a diagnostic tool that enables clinicians to accurately record the maxillary arch dimensions and the size of the alveolar cleft in children with CL/P. This capability is critical during the early phases of treatment planning as it allows for a thorough examination of the oral anatomy and cleft morphology [8,9,10,11]. Another application of DI in cleft care is fabricating the appliances used in pre-surgical orthopaedics, such as NAM appliances [12,13,14,15,16]. DI creates a digital workflow for fabricating appliances that prepare the newborn for cleft lip repair surgery. Furthermore, it is instrumental in evaluating the outcome of various treatments in patients with CL/P [17,18,19,20].

In the pursuit of improving the precision, efficiency, and safety of obtaining oral impressions in neonates with CL/P, DI technology has emerged as a promising alternative [21,22,23]. Incorporating intraoral scanners (IOSs) for DI in CL/P patients overcomes issues associated with traditional procedures. It provides benefits, including instant modifications, decreased discomfort, and the possibility of more efficient operations [16,17]. While the clinical implications of DI in the clinical care of neonates with CL/P have been explored, a comprehensive understanding of the accuracy of these DIs in neonates with CL/P is limited [23]. Moreover, neonates, especially those with CL/P, present a unique challenge due to their small oral cavities, limited cooperation, and potential difficulty in maintaining proper positioning during the scanning process.

This study aimed to assess the accuracy of DIs in neonates with CL/P compared to conventional alginate and putty impressions using a laboratory-based investigation. This study evaluated the accuracy of DIs in this population to determine whether this technology is viable and the potential of streamlining the treatment process and improving patient outcomes. The null hypothesis postulated that there are no significant differences between digital and conventional impressions in neonates with cleft lip and/or cleft palate (CL/P), irrespective of the cleft’s severity (cleft size) and cleft types (Unilateral/Bilateral/Midline Defect).

## 2. Materials and Methods

Ethical approvals for this laboratory-based study were obtained from the Queensland Children’s Hospital Human Research Ethics Committee and from the ethical committee at Griffith University, Queensland, Australia [HREC/23/QCHQ/97036; 3 May 2023].

The inclusion criteria included neonates with cleft lip and/or cleft palate (CL/P), of both genders, with unilateral, bilateral, or midline defects of varying severity. Neonates who required impressions for either NAM or feeding plates were included in this study. The patient models selected consisted of those with a bilateral complete cleft lip and palate, unilateral complete cleft lip and palate, cleft palate only, unilateral cleft of the alveolar ridge only, and unilateral cleft lip only. These patients attended Queensland Children’s Hospital for the management of their cleft defect.

### 2.1. Cleft Lip and Palate Models

**Figure 1:**
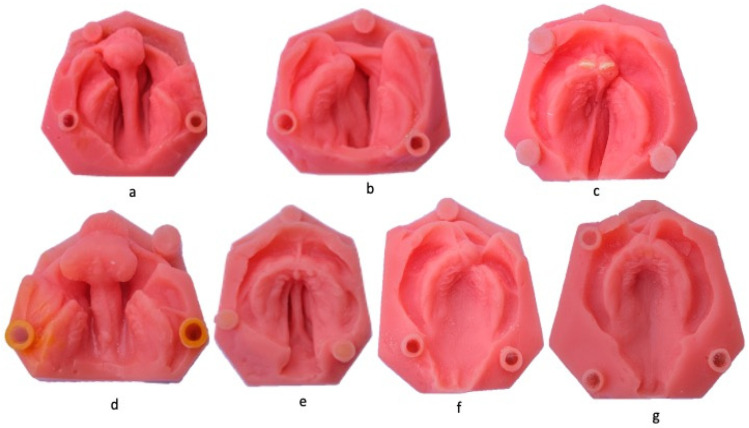
Soft Acrylic models of CL/Ps of varying severity and types: (**a**,**d**) bilateral complete cleft lip and palate; (**b**,**c**) unilateral complete cleft lip and palate; (**e**) cleft palate only; (**f**) unilateral incomplete cleft lip and palate; and (**g**) cleft lip only.

Soft acrylic models were prepared in the Queensland Children’s Hospital (Children’s Oral Health Service and Child Specialist Services, Metro North Hospital and Health Service) for educational and demonstration purposes (Figure 1). These models were duplicated from the original models using a soft acrylic material (Vertex Dental by 3D Systems, Soesterberg, The Netherlands) for the palatal, alveolar pad, and lip areas to mimic the flexibility and softness of the oral tissues. The base of the models was made of hard acrylic (Vertex Rapid, Vertex Dental by 3D Systems, Soesterberg, The Netherlands). Three cylindrical-shaped hard acrylic areas were incorporated into the soft acrylic model’s hard base as a superimposition reference. The purpose of these cylindrical areas was to create a reference point that would not be affected by the impression procedures due to its position outside the area of interest and its hard consistency. Before impression procedures, a cross was delineated in each cylindrical reference to facilitate the selection of multiple reference points for enhanced digital image superimposition. The models included in this study represented a range of cleft conditions: a bilateral complete cleft lip and palate (n = 4), unilateral complete cleft lip and palate (n = 4), unilateral incomplete cleft lip and palate (n = 2), cleft palate only (n = 2), and cleft lip only (n = 2).

### 2.2. Impressions Procedures

#### 2.2.1. Conventional Impressions

To ensure optimal pressure for documenting the intricate details of the oral structures within the alveolar cleft, a customised acrylic tray was designed to enclose the models, including the hard spheres, completely. Alginate impressions (Blueprint XCreme: Dust free Alginates—DENTSPLY Sirona, Charlotte, NC, USA) of the models were obtained using a customised impression tray. The rubber-based impression was obtained using heavy body impression material (Henry Schein V-Posil Mix Putty Fast Set Kit, VOCO-, Cuxhaven, Germany) in a customised impression tray. Inadequate impressions were repeated. Plaster models were generated by filling alginate and rubber-based impressions with dental stone (FUJI ROCK EP Pastel Yellow Die Stone, Gc corporation, Tokyo, Japan) as soon as the impressions were taken.

#### 2.2.2. Digital Impressions

Each soft acrylic model was scanned using a 3Shape TRIOS 4 intraoral scanner (3Shape Dental Systems, Copenhagen, Denmark), which has a smaller scanner head compared to most of the other intraoral scanners on the market, making it more compact, with dimensions of 4.9 × 4.0 × 27.8 cm. Utilising the same IOS, plaster models derived from CIs were scanned and stored as Stereolithographic (STL) images. The scanner head was calibrated daily prior to use to ensure accuracy and reliability. A standardised scanning protocol was meticulously developed to ensure consistency and thoroughness in scanning models with discontinuous arches due to cleft conditions. Firstly, the scan began at one tuberosity area, starting from the greater segment in unilateral cleft cases, setting a starting point for a systematic approach. Then, it extended into the hard acrylic, capturing superimposition spheres to facilitate the precise alignment and merging of scans. Then, it moved to the opposite tuberosity area, ensuring both posterior ends of the arch were comprehensively documented. The process then continued to the palatal surface of the alveolar cleft and then to the anterior part of the alveolar arch. The next step involved scanning the palatal surface of the alveolar arch on the opposite side, providing a detailed view of the upper dental arch’s interior. Finally, the scanning concluded with a focus on the deep part of the cleft, moving from the anterior to the posterior, to ensure a detailed documentation of the cleft’s depth and morphology. A number of inadequately scanned model regions were revisited until the investigator was satisfied with the quality of the scan.

### 2.3. Model Comparison Procedure

This study focused exclusively on assessing trueness while evaluating the accuracy of DIs, as it measures the closeness of a scan’s average dimensions to the true dimensions of an object, providing a direct reflection of the scanner’s ability to replicate the precise morphology of dental structures. The soft acrylic models and the plaster models generated from both alginate and putty impressions were scanned to generate STL files and a comparison was made between the three models

**Figure 2:**
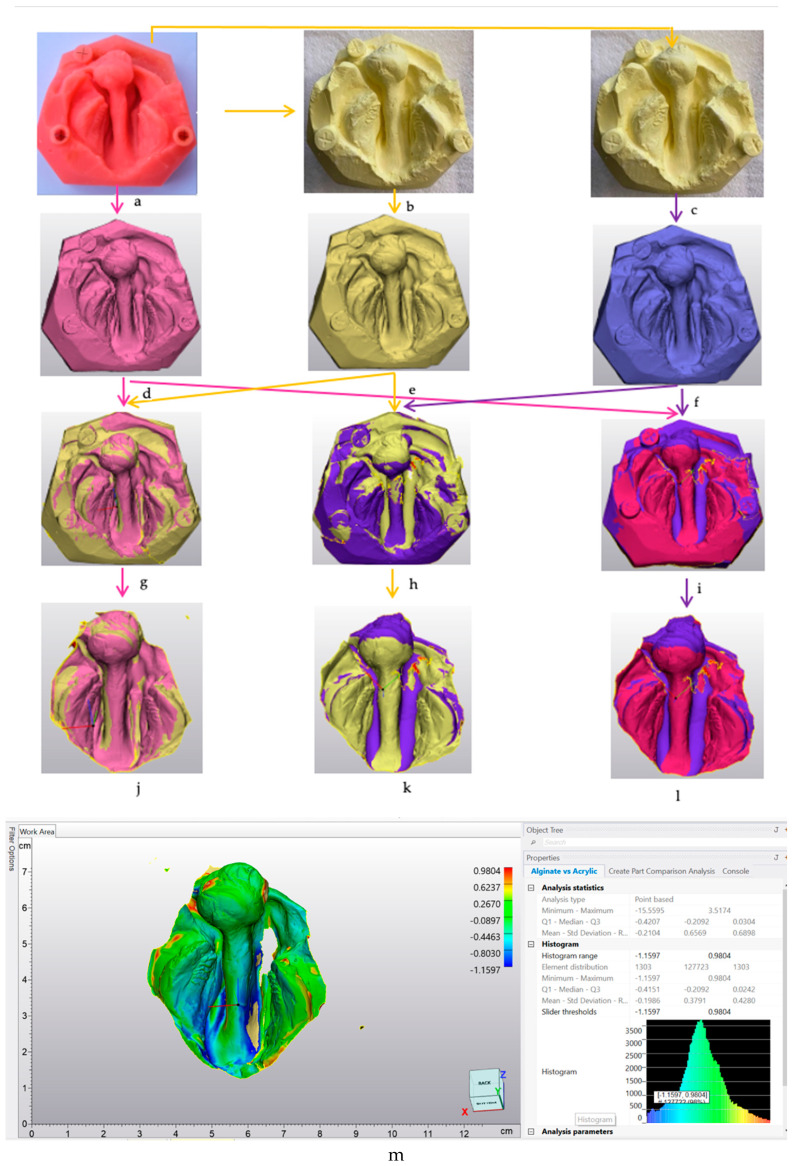
Diagram illustration of the lab-based study: (**a**) soft acrylic model, (**b**,**c**) plaster models generated from alginate and putty impressions, respectively, (**d**–**f**) scanned models from an IOS, (**g**–**i**) superimposed models of alginate vs. IOS, putty vs. IOS, and alginate vs. putty, (**j**–**l**) selected areas of superimposed models showing areas of interest, and (**m**) part comparison of models to m assess surface discrepancies.

Random numbers were assigned to the 3D models of the soft acrylic and plaster models derived from alginate and putty impressions to mitigate potential bias during the data collection process. Using Materialise 3 Matic software Version 16.0 (Materialise, Technologielaan, Leuven, Belgium), three STL images from each group were aligned with N point registration by placing multiple points in the cross mark of the reference sphere. Subsequently, the images were superimposed. Intra-arch measurements and surface discrepancies were compared to analyse the differences among the groups (Figure 2).

#### 2.3.1. Intra-Arch Measurements

**Figure 3:**
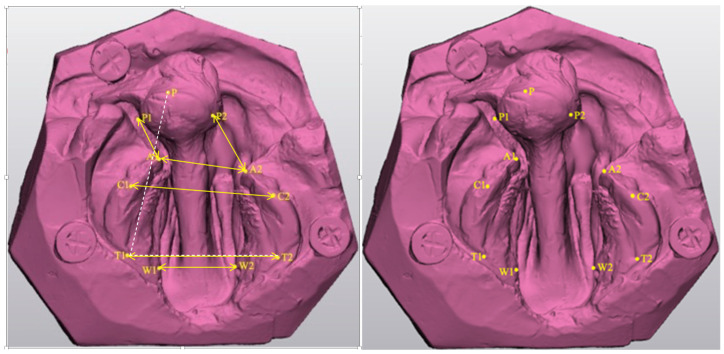
Reference points and intra-arch measurements that were recorded using Materialise 3-Matic software.

Ten anatomical reference points were identified and marked on the superimposed models of a scan (Figure 3). The linear distance, in millimetres, and angular deviations between the reference points were measured from each scan using Materialise 3-Matic software (Materialise, Technologielaan, Leuven, Belgium) (Figure 3). Marking the reference points on the aligned models ensured that the reference points were marked in these exact locations in all images, and it enabled the accurate recording of the linear and angular measurements in all three scanned images (Table 1). Each measurement was repeated three times, and average data values were used to avoid subjective errors. No reliability tests were performed for these measurements. The decision to rely on average values from three repeated measurements was intended to reduce the variability and potential subjective errors inherent in individual measurements.

#### 2.3.2. Surface Discrepancy

The digital scans were exported into Materialise 3-Matic software Version 16.0 (Materialise, Technologielaan, Leuven, Belgium). Scanned images (n = 3) were superimposed in each of the fourteen groups (IOS vs. alginate, IOS vs. putty, and alginate vs. putty), and the surface discrepancy between the three models was analysed. The median values and minimum and maximum discrepancy ranges were documented from histogram data (Figure 2).

### 2.4. Statistical Analysis

A sample size calculation was performed using G*Power software (v3.1.9.2). The calculation assumed a large effect size (d = 0.6), an alpha level of 0.05, and a study power of 80%. The minimum difference considered significant was 0.5 mm. The minimum sample size required was 30 models, with 10 models in each group. Only seven soft acrylic models were available, which were created from the models of seven neonates; to create an adequate sample size, each model was used twice, resulting in a sample size of fourteen models per group. Despite using only seven silicone models, impressions were taken twice to increase the sample size and power of the study. The inherent variability in impression-taking, such as differences in the amount of pressure applied or minor deviations in technique, can indeed result in different outcomes even when the same model is used. Also, the ability to use models multiple times and still obtain reliable data reflects real-world conditions, where clinicians and laboratory staff often work with varying levels of precision and repeatability. The linear and angular measurements were repeated after one month to determine intra-examiner reliability.

An ANOVA test was applied to compare the mean linear and angular measurements between the three groups. The intra-correlation coefficient was calculated to analyse the intra-examiner variability in linear and angular measurements. Data analysis was conducted using IBM SPSS version 29.0. The level of significance was set at a *p* value of <0.05.

## 3. Results

### 3.1. Intra-Arch Measurements

A descriptive statistics overview of many variables’ properties across various parameters is available in Table 2. The variability observed in different dimensions is in line with what is expected in a population with cleft lip and palate, as this naturally includes a range of anatomical variations in size and severity.

An ANOVA test was performed to evaluate the variance distribution between and within groups for each tested variable (Table 3). There was no significant difference (*p* > 0.05) between groups in the PP1-A1, PP2-A2, A1-A2, C1-C2, T1-T2, W1-W2, and P-T1-T2 measurements. The between-groups variance was relatively low, indicating similar measurements across the groups, suggesting no significant differences between groups.

**Figure 4:**
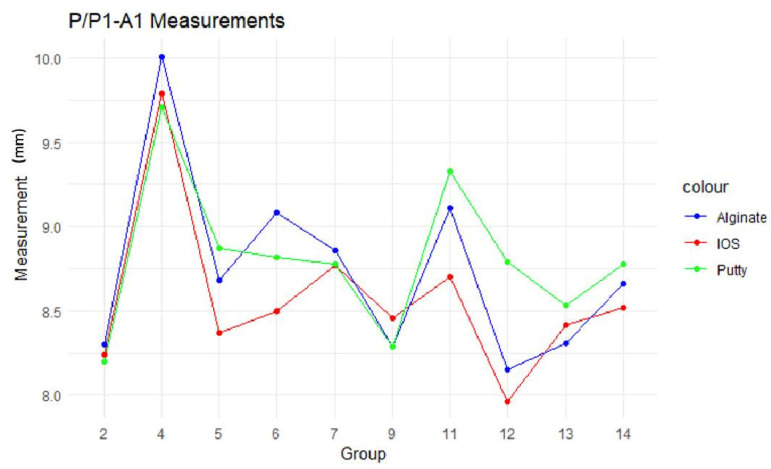

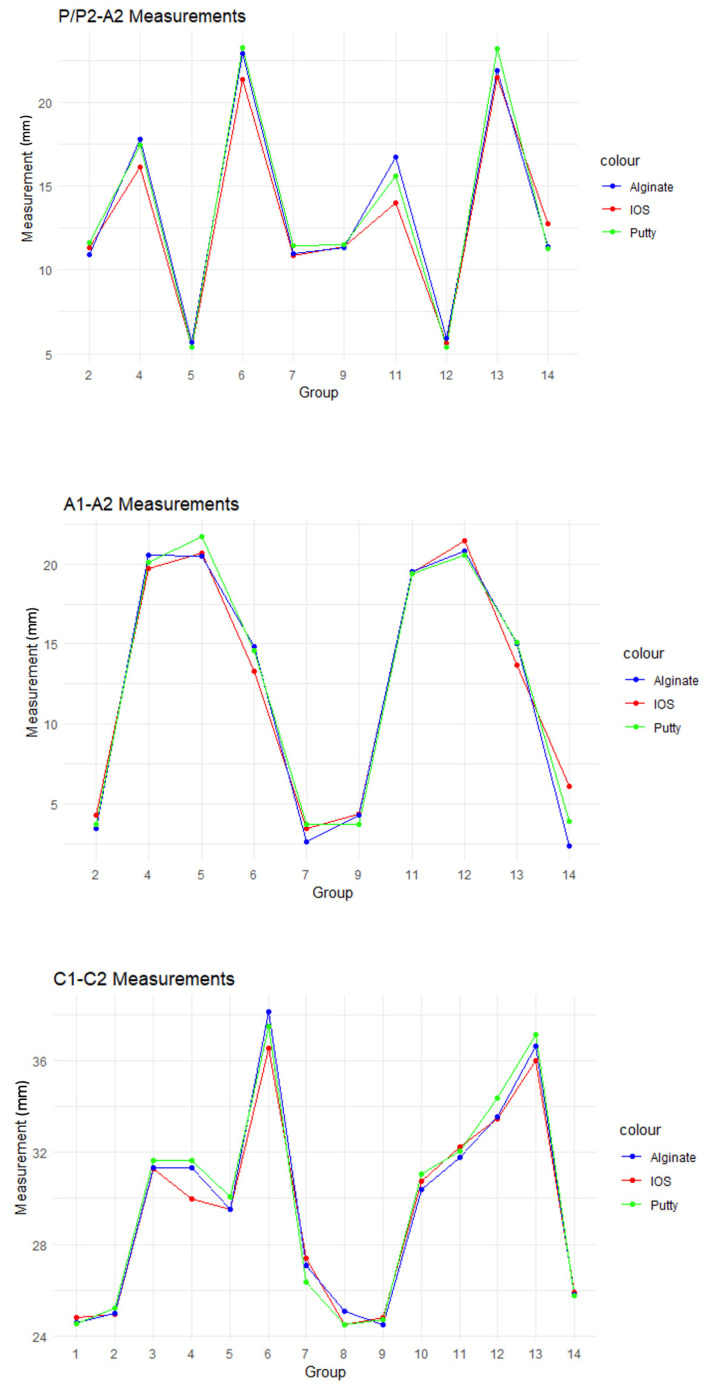

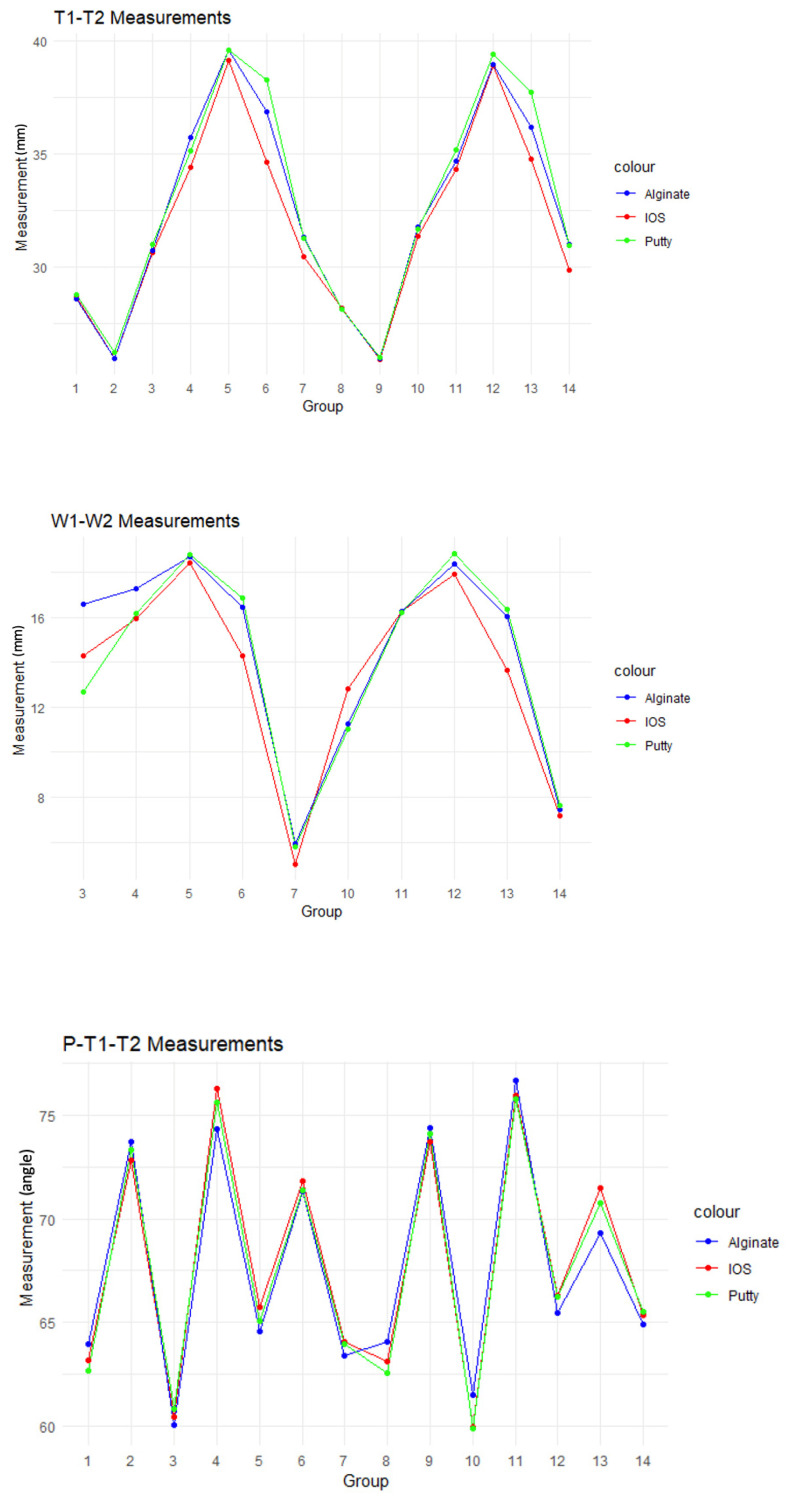
Line graphs representing intra-arch measurements.

For each of the 14 groups of digital models, a line graph represents six linear and one angular measurement for the alginate (Alg), IOS, and putty models (Figure 4). Most groups have a significant degree of similarity in their measurements since the coloured lines that depict each group’s putty, IOS, and alginate are almost precisely parallel. P/P1-A1 had the maximum measurement variation. Still, the degree of deviation was not statistically significant.

### 3.2. Surface Discrepancy

**Figure 5:**
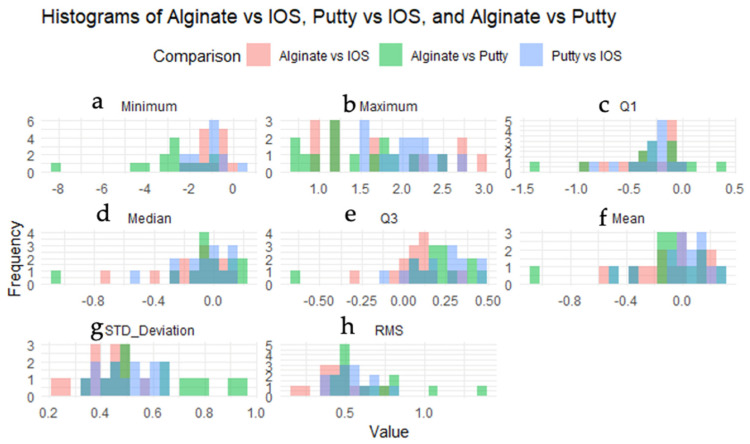
Histogram illustrating the surface discrepancy between different impression methods: alginate vs. IOS (Intraoral Scanner), putty vs. IOS, and alginate vs. putty. The statistical descriptors shown are (**a**) Minimum (Min): the smallest value observed in the dataset, (**b**) Maximum (Max): the largest value observed in the dataset, (**c**) first quartile (Q1): the value below which 25% of the data fall, (**d**) median: the middle value of the dataset, dividing it into two equal halves, (**e**) third quartile (Q3): the value below which 75% of the data fall, (**f**) mean: the average of all the values in the dataset, (**g**) standard deviation (STD): a measure of the amount of variation or dispersion in the dataset, and (**h**) root mean square (RMS): a statistical measure of the magnitude of a varying quantity.

Histograms showed a trend towards negative minimum values for alginate compared to IOS, while positive values for putty suggest that the IOS typically has lower minimum values than putty. This indicates a difference in statistical measurements in the minimum value between the two comparisons (Figure 5a). This study showed a notable difference in the distribution of maximum values between alginate and IOS. Conversely, the maximum values of the putty impression were distributed more uniformly across the dataset compared to those of the alginate and IOS (Figure 5b). However, the first quartile values are primarily negative for alginate and positive for putty, suggesting that alginate typically has lower Q1 values (Figure 5c). The distribution is balanced above and below zero, indicating that the highest values of these materials may not follow a similar trend. The median-value histograms show that alginate and putty have similar medians, with higher median values than IOS (Figure 5d). Their third quartile (Q3) values are greater than the IOS’s (Figure 5e). Alginate has a lower mean value than the IOS but higher than putty (Figure 5f). Standard deviation histograms show that it has higher variability than IOS but lower variability than putty (Figure 5g). RMS histograms show that alginate has a similar RMS to putty and a generally greater RMS than IOS (Figure 5h).

**Figure 6:**
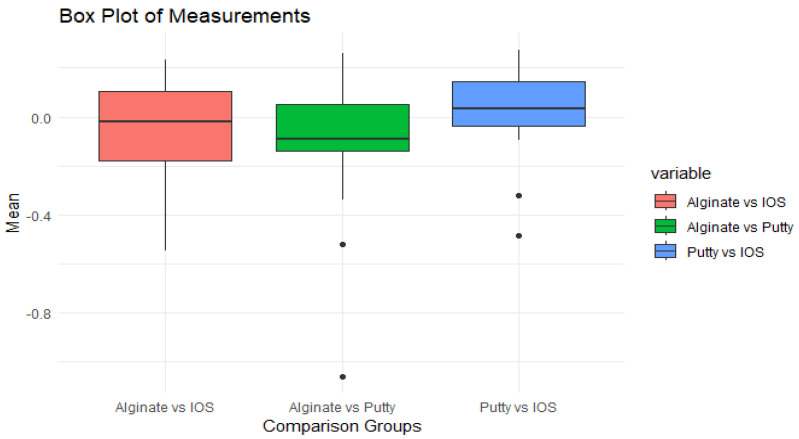
Box plot comparing the surface discrepancy in millimetres between alginate vs. IOS, putty vs. IOS, and alginate vs. putty, highlighting the median, interquartile range, and outliers for each comparison.

Similar observations are made in the box plot of the measurements, showing the mean in the three-comparison group (Figure 6).

The data show a moderate distribution of alginate vs. IOS values, with a median of −0.2 and an interquartile range of −0.3 to +0.1. The outliers below indicate lower alginate values. The data are moderately distributed within this range. Putty vs. IOS has a median value of 0, with noticeable disparities. Alginate vs. putty has less fluctuation, with a narrow IQR and outliers. Most data points lie within a larger range but within 1.5 times the IQR.

The ANOVA table indicates no statistically significant differences in the median and mean values among the groups (Table 4). There are notable variations in the minimum and maximum values across the comparison groups, with *p*-values of 0.000 and 0.026, respectively. The F-statistics are relatively low, and the corresponding *p*-values exceed the conventional significance threshold of 0.05 for variables such as Q1, median, Q3, mean, and RMS. This suggests that there is uniformity in these characteristics and no noticeable variations in averages among the groups for these variables.

Intra-examiner reliability was assessed using Intra Class Coefficient (ICC) estimates and their 95% confidence intervals, which were calculated using IBM SPSS version 29.0 (SPSS Inc., Chicago, IL, USA) based on a mean-rating (*k* = 2), absolute-agreement, and a two-way random model (Table 5). The examiner demonstrated that intra-examiner reliability was high across all three tools (IOS, Alg, putty) at baseline and the one-month follow-up assessments. The measurements made by the examiner are highly consistent, with ICC values close to 1 and statistically significant *p*-values, indicating excellent reliability in the measurements over time.

## 4. Discussion

This study evaluated the trueness of DIs in neonates with CL/P. Trueness and precision are two key components of accuracy in DIs [24]. Trueness refers to the closeness of the average measurement to the true or reference value, essentially assessing how accurately the DI reflects the actual anatomical structure. On the other hand, precision measures the consistency and repeatability of measurements, indicating the degree of variation between repeated measurements of the same object. The primary focus of this study was to evaluate the accuracy of DIs in terms of their ability to reproduce the true anatomical features of the oral cavity. In such cases, assessing trueness alone allows researchers to specifically examine how closely the digital models match the actual patient anatomy, providing valuable insights into the overall performance of IOSs, as trueness is often considered the primary indicator of accuracy in DIs, particularly from a clinical perspective.

The results of this study supported the null hypothesis as there were no significant variations between the superimposed models created from the digital impressions and CIs in neonates with cleft lip and palate. This outcome was evident regardless of the cleft severity (cleft size) and cleft types (unilateral/bilateral). The compared means of the three groups (alginate, IOS, and putty) had no significant differences in either type of measurements; the intra-arch measurements or surface discrepancies. Due to the different sizes and severity of cleft lip and palate in the models used in the study, there was some fluctuation in the measurements. For all measured variables, the analysis of variance (ANOVA) showed no statical differences among the compared groups. Furthermore, the intra-examiner reliability of the measurements was high, indicating excellent consistency and reliability over time.

Descriptive statistics of the intra-arch measurements provide an overview of many variables’ properties across various parameters. PP1_A1 has a mean score of 8.71 and a standard deviation of 0.49, which suggests that there is not much variation in the sample around the mean. On the other hand, variables with higher standard deviations (5.77 and 7.62, respectively), such as A1_A2 and PP2_A2, indicate that their values are more variable. Significant variations exist in the range of values for each variable, with PP2_A2 exhibiting a broad range from 5.37 to 23.30. Additionally, compared to other variables, the means of C1_C2 and T1_T2 are bigger (29.60 and 32.43, respectively), suggesting that their distributions have a higher central tendency. However, this variation in each measurement is insignificant, as the 14 models included in this study consist of cleft lip and palates of varying sizes and severity.

The analysis of variance (ANOVA) findings shed light on how each variable’s group means differ. The F-statistic values vary from 0.004 to 0.13 for all variables (PP1_A1, PP2_A2, A1_A2, C1_C2, T1_T2, W1_W2, and P_T1_T2). Their corresponding *p*-values consistently surpass the traditional significance threshold of 0.05. This suggests no discernible variation between the group means for any given variable. In particular, the *p*-values for variables such as PP1_A1, A1_A2, C1_C2, T1_T2, W1_W2, and P_T1_T2 are all more than 0.05, indicating that there is no significant difference in the group means. All things considered, these ANOVA findings suggest that there are no statistically significant differences in the means of the groups each variable defines. Moreover, the *p*-value for every variable, aside from PP1_A1 (*p* = 0.555), is closer to 1, meaning there is no difference in any of these measurements between the three groups.

This laboratory-based study aimed to assess the accuracy of DIs compared to CIs in neonates with CL/P. In patients with cleft lip and palate, an oral impression is the first procedure that has been undertaken and has typically been carried out in neonates until adulthood, as the rehabilitation of CL/P is a long-term process [2]. DIs have become increasingly popular in all areas of dentistry due to their numerous advantages over traditional impression techniques, such as increased patient comfort, reduced chairside time, and improved accuracy [25]. IOSs are rapidly becoming a favoured tool in paediatric dentistry, especially for improving patient comfort and experience during dental impressions. Recent studies have illuminated the advantages of DI methods over traditional alginate impressions, particularly for children and adolescents [26,27,28,29]. Additionally, IOSs demonstrate comparable accuracy to conventional impression methods in capturing the dental arch morphology in children despite potential challenges such as interference and the inability to produce exact replicas of the dentition [29,30]. However, when it comes to patients with CL/P, the accuracy of DIs becomes even more critical due to the complex anatomical variations and unique challenges presented by this population [30].

There has been some growing research on the viability of using IOSs in neonates and infants with CL/P to explore the potential application of these technologies in various stages of CL/P treatment [9,16,22]. However, very few studies have rigorously assessed the accuracy of IOSs in neonates with CL/P, a critical factor for effective treatment planning and outcome evaluations in this group. If the DI is accurate and efficient, replacing CIs with digital ones may remarkably reduce the risks and discomfort associated with the procedure. Therefore, validating the accuracy of IOSs in neonates with cleft lip and palate patients is a prerequisite for their use in clinical care.

The results of our study substantially contribute to the body of knowledge about the accuracy of DIs compared to CIs produced by putty and alginate in neonates with CL/P. Our findings align with those of Patel (2019), El Naghy (2022), and Okazaki (2023) in highlighting consistent intra-arch measurement characteristics, statistically insignificant differences across variables, and high intra-examiner reliability and offering valuable insights for the use of digital impression in neonates with CL/P [9,31,32]. Patel and El Naghy utilised superimposition exclusively and Okazaki focused on intra-arch measurements, whereas our study adopted a comprehensive approach, integrating both intra-arch measurements and surface discrepancy analyses, offering a broader overview of the accuracy of DIs compared to CIs. Our study’s findings align with research that compared the accuracy of IOSs with alginate and putty materials in edentulous arches, emphasising the accuracy of IOSs in capturing detailed impressions across a variety of dental landscapes, including the challenging scenario of edentulous arches [33].

The line diagrams of the intra-arch measurement variations between the IOS, alginate, and putty impressions presented in our study demonstrate the lack of a consistent pattern in how these distances change, emphasising the differences in material behaviour and dimensional stability when capturing dental impressions. The difference in the measurements was not statistically significant, with the most variation in the PP/P1-A1 measurement. This variation could likely be attributed to the soft, movable anterior segment distortion resulting from the pressure applied by the impression materials. In groups exhibiting greater variation, alginate sometimes showed larger fluctuations, while putty demonstrated more variability in other instances. Despite the observed inconsistencies in linear distance variations between the alginate and putty impressions, the analysis of surface deviations reveals that the variability between putty impressions and intraoral scanning (IOS) is significantly less, indicating a closer agreement in the accuracy of these two methods, as depicted in the box plot. The reduced variability between putty impressions (Polyvinyl siloxane- PVS) and IOSs compared to alginate impressions can be explained by considering the material’s qualities and the effects of syneresis and imbibition on dimensional stability. Todd et al., on the dimensional variations in extended-pour alginate impression materials due to syneresis and imbibition, reveal that alginate tends to undergo dimensional changes over time [34]. The reduced variability between putty impressions and the IOS compared to alginate is due to the specific material qualities and environmental resilience of PVS and digital impressions. Furthermore, the alginate and putty impressions were poured immediately after being made to create the plaster models to minimise dimensional alterations.

The surface discrepancy analysis revealed patterns in the minimum, maximum, median, and standard deviation values between the three groups (alginate, IOS, and putty). Overall, there were no significant differences in the surface discrepancies among the groups. The box plots further illustrated the homogeneity in the differences between the groups. However, the analysis did show statistically significant differences in the minimum and maximum values of the alginate among the groups compared, but not in its median or standard deviation values. While extreme discrepancies may vary, the general accuracy across methods remains consistent. The difference observed in the minimum and maximum values for alginate impressions can be attributed to material-specific behaviours under pressure, as explained by Patel et al. [9]. They noted that the maximum deviation was observed in the freely movable premaxilla, suggesting that the deformation could be due to the pressure exerted by the alginate impression material during the impression-taking process. This could be explained by the thinner consistency of the alginate impression material in comparison to the putty impression material, which allows excessive flow of the material around the freely movable premaxilla even with minimal pressure exerted during the impression-taking process. No constant deviations were observed in specific areas in this investigation, highlighting the intricate interaction of elements that affect impression accuracy. Currently, no studies compare the accuracy of alginate and putty in cleft lip and palate (CL/P) patients and how it varies with material properties.

In the present study, we utilised the Trios 4 scanner, leveraging the advanced capabilities of the Trios 3Shape intraoral digital impression system, which is based on the principles of ultrafast optical sectioning and confocal microscopy. This system employs range finding to accurately define surface manifolds, maintaining a constant spatial relationship between the scanner and the object [35]. Its notable scanning speed of up to 3000 images per second significantly reduces errors due to patient movement, enabling the rapid creation of precise digital 3D models of teeth and arch forms. Scanners with high-speed imaging capabilities and advanced optical technologies, such as confocal microscopy, would be better suited for working with neonates, as they can quickly capture detailed images, reducing chairside time. This is especially important for neonates with CL/P, who may experience discomfort or have difficulty remaining still for long periods.

The scanning strategy we used, considering the suggestion by Weise et al., focused on a comprehensive approach to create the continuity of the scanned areas [36]. It involved starting with the least deformed area to establish a reliable baseline for the scan, progressively moving towards more complex regions. Having soft acrylic models with a rigid base significantly facilitated this scanning strategy by providing a stable foundation that ensured a consistent scan quality, even across the complex and varied topographies characteristic of cleft lip and palate (CL/P) conditions. The rigid base served as a reliable anchor point, allowing for the accurate initiation and stabilisation of the scanning process. With the Trios 4 scanner, we deviated from the ideal strategy specific to this particular device. This strategy ensures that the scanner accurately registers the intricate details of the cleft area by building upon an initial, more stable digital framework. Intra-oral scanning in neonates with cleft lip and palate (CL/P) requires experimenting with various scanning strategies to achieve optimal results. Due to their delicate oral anatomy and difficulty maintaining stillness, flexibility in scanning techniques is crucial to accommodate cleft morphology and severity variations. By systematically testing different strategies, the best scanning strategies can be identified that bring out the optimal balance between accuracy, efficiency, and patient comfort, ultimately enhancing the quality of care and treatment outcomes for neonates with CL/P.

The choice of scanning tip size could influence the accuracy and efficacy of DI, particularly in neonates with cleft lip and palate (CL/P). Larger scanning tips may struggle to navigate the narrow and complex spaces in neonates with CL/P, potentially leading to incomplete or inaccurate DIs, especially in deep cleft areas with limited access. Moreover, a few studies have reported the inability of an IOS to capture the deepest part of the alveolar cleft. Case reports and studies explaining the digital workflow for NAM in neonates with CL/P advocates for small scanning tips. Compact and easy-to-manoeuvre devices are preferable in a neonatal setting, where space is often limited and the patient’s comfort is a priority. Previous studies in adult edentulous dentition show that a smaller scanning tip could compromise the accuracy of the DI [37]. Hence, more studies are recommended in this area to determine how a smaller scanning tip could benefit neonates with CL/P and its impact on the accuracy of DIs.

Our findings must be evaluated in consideration of specific limitations. The findings of this study are limited by its in vitro nature, which may not accurately represent the clinical validity of the impressions acquired. In a case study, Patel et al. found that the premaxillary region of a BCLP is where the difference between a conventional and digital impression occurs. This discrepancy may result from pressure exerted on the freely moving premaxilla during the use of a CI, which might cause deformation. The absence of these typical clinical conditions might have resulted in an idealised experimental environment, potentially leading to more controlled and consistent intra-arch measurements and surface analyses than would be encountered in real-world clinical settings. To compensate for the lack of these clinical settings, we used soft acrylic models intended to resemble the softness of oral tissue. This decision was made to simulate the impression procedures accurately, as soft tissue displacement can occur due to the pressure applied during these procedures. This is particularly critical in cases of bilateral cleft lip and palate (BCLP), where the premaxilla is mobile and the anterior segment can be displaced as the impression material pushes through the cleft defect. Regarding the digital impressions, it is indeed correct that the softness or hardness of the material does not affect the scanning process or results from the IOS. The scanner captures surface data effectively regardless of the material’s properties. However, using soft acrylic models allowed us to better simulate the clinical conditions under which traditional impression materials are used. This ensures that our comparisons between traditional and digital methods are as realistic and clinically relevant as possible. By using these soft acrylic models, we aimed to bridge the gap between experimental conditions and real-world clinical scenarios, enhancing the validity of our findings regarding the accuracy of digital impressions in reproducing true anatomical features. This approach provides a comprehensive understanding of how both traditional and digital impression techniques perform under conditions that closely mimic the clinical scenario. A systematic review reporting the clinician-centred outcomes related to DIs in infants with CL/P reported that clinicians faced some challenges while recording the oral structures in this population, such as frequent head movement, increased salivation, with a preference for a smaller scan tip due to the limited mouth opening which could affect the quality of DIs [23]. Also, the quality of the scanned images can be affected by the scanner’s optical characteristics and the presence of blood and saliva [38]. However, in vitro investigations can aid clinical scenarios by offering a controlled setting and minimising confounding variables [39]. Our study, though conducted in vitro, aligns with accuracy assessments of IOSs in various dental fields, including orthodontics, as supported by systematic reviews such as Aragón et al. and Floriani et al. [40,41,42].

The current study has the limitation of being designed as a lab study with a limited number of cleft severity variations, particularly with the objective of accuracy from the aspect of trueness. Additional lab studies are necessary to explore the optimal scan strategies to achieve the best digital impression outcomes in cleft lip and palate infants. It is crucial to validate the accuracy of digital impressions through clinical studies to confirm the current findings. These aspects are under research by the ADD Tec+ research group at Metro North Health and Queensland Children’s Hospital in Brisbane, Australia.

## 5. Conclusions

In conclusion, our study demonstrates that DI exhibits comparable accuracy to CI techniques in neonates with cleft lip and palate (CL/P). Through a systematic evaluation of trueness, we have shown that DIs reliably capture the intricate anatomical details of the oral cavity in this vulnerable patient population. These findings suggest that DI holds promise as a viable alternative to CI methods for neonates with CL/P, offering the potential for improved efficiency, patient comfort, and treatment outcomes. While further research is needed to fully explore the clinical implications of DI and the factors that could affect it, our study provides valuable evidence supporting its feasibility and accuracy in managing CLP in neonates.

## Figures and Tables

**Table 1:** Reference points and measurements.

Reference Points	
P	Midline of the premaxilla
P1 and P2	The most prominent inferior portion of the premaxilla bilaterally
A1 and A2	The most prominent points in the anterior portion of the cleft bilaterally
C1 and C2	The canine points bilaterally
T1 and T2	The maxillary tuberosities bilaterally
W1 and W2	The most prominent points in the posterior portion of the cleft bilaterally
Linear and Angular Measurements	
P1-A1 and P2-A2or P-A1 and P/A2	The distance between the premaxilla and the most anterior point of the alveolar cleft
A1-A2	The linear distance measured around alveolar ridges anteriorly
C1-C2	Inter canine distance
T1-T2	Inter tuberosity distance
W1 and W2	Posterior cleft width, linear distance measured between the most prominent points in the posterior part of the cleft on the right and left sides
P-T1-T2	The deviation angle from the inter tuberosity line to the most anterior part of the premaxilla

**Table 2:** Descriptive statistics of the linear and angular measurements.

Descriptive Statistics					
	N	Minimum	Maximum	Mean	Std. Deviation
PP1-A1 (mm)	30	7.96	10.01	8.7093	0.49249
PP2-A2 (mm)	30	5.37	23.30	13.4010	5.76788
A1-A2 (mm)	30	2.35	21.74	12.5697	7.61835
C1-C2 (mm)	42	24.48	38.14	29.6019	4.32987
T1-T2 (mm)	42	25.91	39.60	32.4302	4.40571
W1-W2 (mm)	30	5.01	18.84	14.0090	4.34986
P-T1-T2 (angle)	42	59.85	76.71	67.7481	5.44689
Valid N (listwise)	24				

**Table 3:** ANOVA statistical results for all groups’ differences in their linear and angular measurements, including the Sum of Squares (measured in mm), degrees of freedom (df), mean square (measured in mm), F-values, and significance levels (Sig.).

ANOVA						
		Sum of Squares (mm)	df	Mean Square (mm)	F	Sig.
PP1_A1	Between Groups	0.300	2	0.150	0.601	0.555
	Within Groups	6.734	27	0.249		
	Total	7.034	29			
PP2_A2	Between Groups	2.026	2	1.013	0.028	0.972
	Within Groups	962.758	27	35.658		
	Total	964.784	29			
A1_A2	Between Groups	0.442	2	0.221	0.004	0.996
	Within Groups	1682.695	27	62.322		
	Total	1683.137	29			
C1_C2	Between Groups	0.705	2	0.353	0.018	0.982
	Within Groups	767.954	39	19.691		
	Total	768.659	41			
T1_T2	Between Groups	5.402	2	2.701	0.133	0.876
	Within Groups	790.419	39	20.267		
	Total	795.821	41			
W1_W2	Between Groups	3.654	2	1.827	0.091	0.914
	Within Groups	545.064	27	20.188		
	Total	548.718	29			
P_T1_T2	Between Groups	0.323	2	0.162	0.005	0.995
	Within Groups	1216.088	39	31.182		
	Total	1216.411	41			

**Table 4:** ANOVA results for the differences in surface discrepancy variables between groups. This table includes the Sum of Squares (measured in mm), degrees of freedom (df), mean square (measured in mm), F-values, and significance levels (Sig.). The key statistical measures analysed are the minimum, maximum, first quartile (Q1), median, third quartile (Q3), mean, standard deviation (SD), and root mean square (RMS).

ANOVA						
		Sum of Squares (mm)	df	Mean Square (mm)	F	Sig.
Minimum	Between Groups	32.260	2	16.130	11.090	0.000
	Within Groups	56.726	39	1.455		
	Total	88.986	41			
Maximum	Between Groups	2.618	2	1.309	4.003	0.026
	Within Groups	12.754	39	0.327		
	Total	15.372	41			
Q1	Between Groups	0.001	2	0.001	0.005	0.995
	Within Groups	4.034	39	0.103		
	Total	4.035	41			
Median	Between Groups	0.038	2	0.019	0.319	0.729
	Within Groups	2.315	39	0.059		
	Total	2.353	41			
Q3	Between Groups	0.129	2	0.064	1.625	0.210
	Within Groups	1.548	39	0.040		
	Total	1.677	41			
Mean	Between Groups	0.152	2	0.076	1.140	0.330
	Within Groups	2.607	39	0.067		
	Total	2.759	41			
S.D	Between Groups	0.275	2	0.138	7.523	0.002
	Within Groups	0.713	39	0.018		
	Total	0.988	41			
RMS	Between Groups	0.339	2	0.170	4.599	0.016
	Within Groups	1.438	39	0.037		
	Total	1.777	41			

**Table 5:** Intra-examiner reliability using Intra Class Coefficients, demonstrating high reliability.

Group	Time	Mean	Intraclass Correlation	95% Confidence Interval		*p* Value
				Lower Bound	Upper Bound	
IOS	Baseline	28.97	1.000	0.997	1.000	<0.001 *
	1 Month	29.09				
Alg	Baseline	29.206	0.999	0.997	1.000	<0.001 *
	1 Month	29.358				
Putty	Baseline	29.271	0.999	0.997	1.000	<0.001 *
	1 Month	29.395				

*: *p* < 0.001.

## Data Availability

The work includes all the original contributions made, and any additional questions can be forwarded to the corresponding author.
